# Patterns of digital device usage and service needs for digital adaptation among older adults

**DOI:** 10.1093/geroni/igaf122.2811

**Published:** 2025-12-31

**Authors:** Sang A Lee, Eunhee Cho, Minhee Yang, Jungwon Cho, Eun Kyo Kim, Sinwoo Hwang

**Affiliations:** Yonsei University, Seoul, Republic of Korea; Yonsei University, Seoul, Republic of Korea; Yonsei University, Seoul, Republic of Korea; Yonsei University, Seoul, Republic of Korea; Yonsei University, Seoul, Republic of Korea; Yonsei University, Seoul, Republic of Korea

## Abstract

In the digital era, digital devices play an increasingly vital role in health management beyond daily convenience. However, age-related physical changes and low digital literacy hinder older adults’ technology use, widening the digital divide and impacting health. This study analyzed subgroups of older adults based on digital device usage and difficulties in digital adaptation to identify their service needs. A total 7,470 older adults were sampled from the 2023 National Survey of Older Koreans. Latent class analysis classified digital usage subgroups using 13 digital device activities (e.g., text messaging, social media, information search, photos/videos, music, games, and financial transactions) and one item on digital adaptation difficulties. Multinomial logistic regression analyses were performed to examine socio-demographic and health-related factors of each subgroup and to identify their preferred service needs. Four subgroups emerged from the analysis: digitally active (DA) (14.9%), moderately active (MA) (33.4%), text messages only (TM) (27.9%), and digitally inactive (DI) (23.9%). Compared to the DA group, the DI and TM groups were older, had more chronic diseases, lower cognitive function, and required greater assistance in daily life. Regarding service needs, compared to the DA, the other groups showed a stronger preference for age-friendly technology support (e.g., larger fonts, simplified layouts) over digital literacy education (DI: OR = 2.065, 95% CI = 1.603–2.662; TM: OR = 1.568, 95% CI = 1.259–1.953; MA: OR = 1.331, 95% CI = 1.095–1.618). By identifying subgroup-specific needs, this study provides policy and practical insights to develop targeted interventions, ensuring older adults receive the necessary support to navigate the evolving digital landscape.

